# Prognostic implications of dynamic serum lactate dehydrogenase assessments in nasopharyngeal carcinoma patients treated with intensity-modulated radiotherapy

**DOI:** 10.1038/srep22326

**Published:** 2016-03-01

**Authors:** Guan-Qun Zhou, Xian-Yue Ren, Yan-Ping Mao, Lei Chen, Ying Sun, Li-Zhi Liu, Li Li, Ai-Hua Lin, Hai-Qiang Mai, Jun Ma

**Affiliations:** 1 State Key Laboratory of Oncology in Southern China, Collaborative Innovation Center of Cancer Medicine, Department of Radiation Oncology, Cancer Center, Sun Yat-sen University, Guangzhou 510060, People’s Republic of China; 2 State Key Laboratory of Oncology in Southern China, Collaborative Innovation Center of Cancer Medicine, Imaging Diagnosis and Interventional Center, Cancer Center, Sun Yat-sen University, Guangzhou 510060, People’s Republic of China; 3 Department of Medical Statistics and Epidemiology, School of Public Health, Sun Yat-sen University, Guangzhou 510060, People’s Republic of China; 4 State Key Laboratory of Oncology in Southern China, Collaborative Innovation Center of Cancer Medicine, Department of Nasopharyngeal Cancer, Cancer Center, Sun Yat-sen University, Guangzhou 510060, People’s Republic of China

## Abstract

The prognostic value of dynamic serum lactate dehydrogenase (LDH) levels in patients with nasopharyngeal carcinoma (NPC) treated with intensity-modulated radiotherapy (IMRT) hasn’t been explored. We retrospectively analyzed 1,428 cases of NPC treated with IMRT with or without chemotherapy. Elevated pre- and/or post-treatment LDH levels were found to be associated with unfavorable overall survival (OS), disease-free survival (DFS) and distant metastasis-free survival (DMFS), but not with local relapse-free survival (LRFS). The dynamic variations in LDH levels were prognostic factors for OS, DFS and DMFS, but not for LRFS. Multivariate analysis revealed that the N category, T category, post-treatment serum LDH level and age were independent prognostic factors for OS. Our results demonstrated that dynamic variations in LDH levels were associated with risk of distant failure and death, which may shed light on the dynamics of the disease and the response to therapy. We consider that LDH measurements will be of great clinical importance in the management of NPC, especially, when considering “decision points” in treatment algorithms. Therefore, we strongly recommend that LDH levels should be determined before and after treatment in NPC patients and the results integrated into decisions regarding treatment strategies.

Nasopharyngeal carcinoma (NPC) has an extremely unbalanced endemic distribution, and high-incidence areas in southern China have an age-standardized NPC incidence rate of 20–50 per 100,000 males^1^. Historically, radiotherapy has been the mainstay of treatment for achieving local and regional control of this disease^2^. The extent of local invasion, regional lymphatic spread and distant metastasis, as reflected by the TNM stage, are the most widely used parameters to formulate rational treatment strategies and predict clinical outcomes in NPC. However, the current TNM staging system does not take into account the biological variability of the tumor itself, and patients in the same TNM stage can still show substantial clinical heterogeneity^3^. The limited power of TNM staging in determining individual patient outcomes highlights the need for better prognostic indicators for NPC.

Numerous attempts have been made to establish useful systems to predict the survival of NPC patients^4^,^5^. The serum levels of many enzymes and signaling molecules have been identified as useful prognostic factors of individual tumor characteristics, facilitating the evaluation of disease development and treatment effectiveness^4^,^5^. Our previous study showed that the baseline plasma lactate dehydrogenase (LDH) level was a useful tumor marker in NPC management^6^. The baseline LDH level correlates well with tumor stage, outcome prediction and early detection of liver metastasis in patients with NPC, and has been developed as a tool for the non-invasive assessment of the tumor burden in NPC patients^6^,^7^,^8^.

Most scoring systems presented so far use LDH as a “static” prognostic variable determined at the time of diagnosis^8^,^9^, and the clinical significance of dynamic LDH levels measured at different time points has not been fully explored. The dynamics of the disease and responses to therapy may be of great clinical importance, especially when considering “decision points” in treatment algorithms such as residual gross tumor at the end of radiotherapy. Therefore, in this study, we retrospectively reviewed the medical records of patients with newly diagnosed, non-metastatic NPC treated with radical intensity-modulated radiation therapy (IMRT). The predictive and prognostic roles of pre-treatment and post-treatment serum LDH levels were evaluated, particularly with respect to the establishment of prognostic subsets of NPC and appropriate treatment strategies.

## Materials and Methods

### Patient selection

We retrospectively reviewed the records of all NPC patients who were treated with IMRT at the Cancer Center of Sun Yat-sen University (Guangzhou, People’s Republic of China) between November 2009 and February 2012. This study was approved by the institutional review board. We excluded 383 patients because their records contained insufficient information. Thus, we reviewed the cases of a total of 1,428 patients with newly diagnosed, histologically proven, non-metastatic NPC.

### Clinical staging

All patients underwent pretreatment evaluations that included a complete history, physical examinations, hematology and biochemistry profiles, MRI of the nasopharynx and neck, chest radiography, abdominal sonography, and whole-body bone scan using single photon-emission computed tomography. All MRI and clinical data were reviewed to minimize heterogeneity in restaging. Two radiologists specializing in head and neck cancers separately evaluated all scans, and any disagreements were resolved by consensus. The American Joint Committee on Cancer staging system (7th edition) was used for stage classification^10^.

### Treatment

All patients were treated with radical IMRT over the entire course of treatment. Details regarding the IMRT techniques used have previously been reported^11^. In general, the treatment plans were determined according to tumor stage and general health of the patient. During the study period, institutional guidelines recommended radiotherapy alone for patients in stage I, concurrent chemoradiotherapy for those in stage II, and concurrent chemoradiotherapy with or without neoadjuvant/adjuvant chemotherapy for those in stage III to IVb defined by the 7th edition of the UICC/AJCC staging system for NPC. Of the 1,088 patients with stage III or stage IVA-B disease (classified as T3–T4 or N2–N3), 1,034 (95.0%) received chemotherapy, including various regimens of concurrent chemotherapy in combination with either induction chemotherapy or adjuvant chemotherapy in conjunction with a platinum-based therapeutic clinical trial. Concurrent chemotherapy consisted of 80–100 mg/m^2^ every 3 weeks or 40 mg/m^2^ weekly cisplatin. Induction or adjuvant chemotherapy consisted of 2–4 cycles of cisplatin with 5-luorouracil (80 mg/m^2^ cisplatin on day 1 and 800 mg/m^2^ per day 5-fluorouracil on days 1–5), or cisplatin with docetaxel every 3 weeks (70 mg/m^2^ cisplatin and 70 mg/m^2^ docetaxel on day 1), or cisplatin with 5-luorouracil and docetaxel (60 mg/m^2^ cisplatin on day 1, 60 mg/m^2^ docetaxel on day 1 and 600 mg/m^2^ per day 5-fluorouracil on days 1–5). When possible, salvage treatments, including afterloading, surgery and chemotherapy, were provided in the event of a documented relapse or persistent disease.

### Patient follow-up

Serum LDH levels were measured within 14 days of any therapeutic intervention (with the exception of diagnostic biopsy of primary tumors or cervical lymph nodes) and within 4 weeks after the completion of treatment. The specific procedures of LDH analysis have been described in our previous study^6^. Normal serum LDH enzyme activities ranged from 109–245 IU/l, and the coefficient of variance for the LDH measurements was <5.0%.

Patients returned for follow-up appointments at least every 3 months during the first 2 years and every 6 months thereafter, until death. The follow-up duration was calculated from the first day of therapy to the day of death or to the day of the last examination. The following end points (time to the first defining event) were assessed: overall survival (OS), disease-free survival (DFS), distant metastasis–free survival (DMFS) and local relapse–free survival (LRFS).

### Statistical analysis

The Statistical Package for the Social Sciences, version 16.0 (SPSS, Chicago, IL) was used for all statistical analyses. The independent-samples *t*-test was used to calculate differences in continuous variables between the various patient groups. The pre-treatment and post-treatment serum LDH levels were compared using paired-samples *t*-tests. Actuarial rates were calculated using the Kaplan–Meier method, and differences were compared using the log-rank test. Multivariate analysis using a Cox proportional hazards model was used to test for independent significance by backward elimination of insignificant explanatory variables. Host factors (including age, sex, pathology and clinical stage) were included as covariates in all tests. The criterion for statistical significance was set at α = 0.05 and *P*-values were based on two-sided tests.

## Results

### Patient characteristics

The characteristics of the included patients have been listed in Table 1. Among the 1,428 study patients, 1,066 were male and 362 were female (male/female ratio, 2.94:1). The median patient age was 44.0 years (range, 14–78 years). On histological examination, 99.5% of the patients were diagnosed with type II or III disease, according to the guidelines of the World Health Organization (WHO)^12^. Stage I, II, III and IVA-B NPC was present in 4.4%, 19.4%, 47.9% and 28.3% of the study patients, respectively. The median follow-up period was 44.7 months (range, 3.1–67.5 months).

Locoregional failure or distant metastases were detected in 16.4% (234/1,428) patients, and 6.9% (99/1,428) patients did not survive. In the entire cohort, the 4-year OS, DFS, DMFS and LRFS rates were 90.9%, 82.1%, 88.9% and 91.7%, respectively. The 4-year OS rates for patients in stages I, II, III and IV were 100%, 96.3%, 92.6% and 83.3%, respectively.

### LDH level at diagnosis and its prognostic significance

At the time of diagnosis, 93.6% (1,337/1,428) of the NPC patients had serum LDH levels within the normal limits (i.e., 109–245 IU/l). The remaining 6.4% (91/1,428) patients exhibited elevated serum LDH levels. The median and mean pre-treatment serum LDH levels for the entire cohort were 170.00 IU/l and 178.06 ± 2.28 IU/l, respectively (range, 84–453 IU/l). The pre-treatment LDH levels progressively increased with worsening clinical stage, and the mean LDH values at diagnosis in patients with stage I–IV disease were 162.16 ± 27.78 IU/l, 168.94 ± 35.31 IU/l, 176.34 ± 53.96 IU/l and 186.09 ± 54.38 IU/l, respectively (*P* < 0.001). Of the 91 patients with elevated serum LDH levels at the time of diagnosis, 12 (13.2%) had stage II disease, 37 (40.7%) had stage III disease and 42 (46.2%) had stage IV disease.

The 4-year OS rate among the patients with normal pretreatment serum LDH levels (90.9%) was significantly higher than the rate in patients with elevated LDH levels (80.0%; *P* = 0.001). Significant differences between patients with normal and elevated baseline LDH levels were also detected in the case of the 4-year DFS (83.4% and 62.3%; *P* < 0.001) and DMFS (90.1% and 70.5%; *P* < 0.001). However, the 4-year LRFS was the same regardless of the LDH value at diagnosis (91.7% and 90.8%; *P* = 0.61).

### Post-treatment LDH levels as a prognostic parameter

After treatment, 97.5% (1,392/1,428) of the NPC patients had normal serum LDH levels, and 2.5% (36/1,428) of the patients exhibited elevated serum LDH levels. The median and mean post-treatment serum LDH levels were 153.00 IU/l and 159.39 ± 1.50 IU/l, respectively. The paired-samples *t*-test revealed that the LDH level significantly declined after treatment (*P* < 0.001). No differences were found in post-treatment LDH levels between patients in different clinical stages (*P* = 0.673), and the mean post-treatment LDH levels in patients with stage I–IV disease were 154.08 ± 27.86 IU/l, 156.78 ± 39.27 IU/l, 159.74 ± 79.31 IU/l and 160.94 ± 36.97 IU/l, respectively.

**Figure 1:**
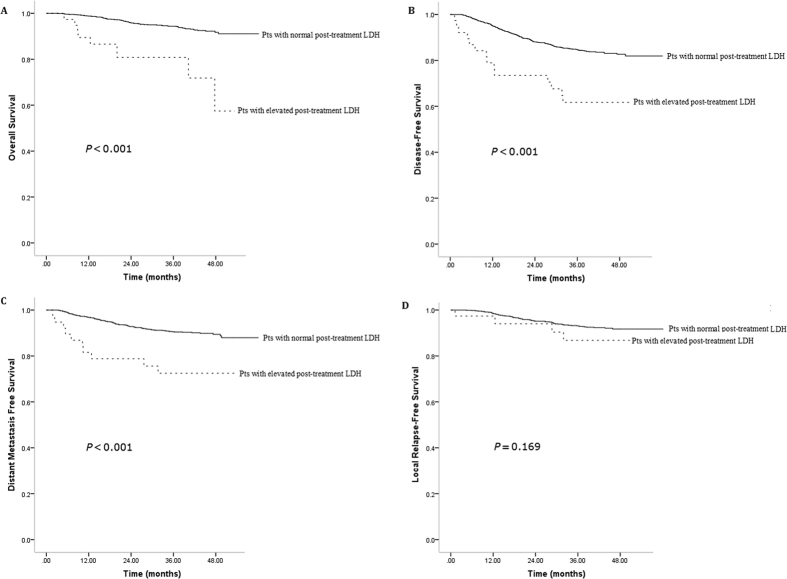
Kaplan–Meier survival curves showing overall survival rates (**A**), disease-free survival rates (**B**), distant metastasis–free survival rates (**C**) and local relapse–free survival rates (**D**) in nasopharyngeal carcinoma patients with elevated or normal post-treatment levels of lactate dehydrogenase (LDH).

The post-treatment LDH levels were identified as a statistically significant prognostic factor for OS (*P* < 0.001). The 4-year OS of patients with elevated and normal LDH levels after treatment was 91.7% and 60.6%, respectively (Fig. 1A). Patients with normal post-treatment LDH also had significantly better 4-year DFS (82.6% vs. 64.2%; *P* = 0.001; Fig. 1B) and DMFS (89.3% vs. 73.2%; *P* < 0.001; Fig. 1C). In contrast, there was no prognostic impact of post-treatment serum LDH levels on LRFS (91.8% vs. 86.2; *P* = 0.169; Fig. 1D).

### Dynamic variation in pre- and post-treatment serum LDH levels

**Figure 2:**
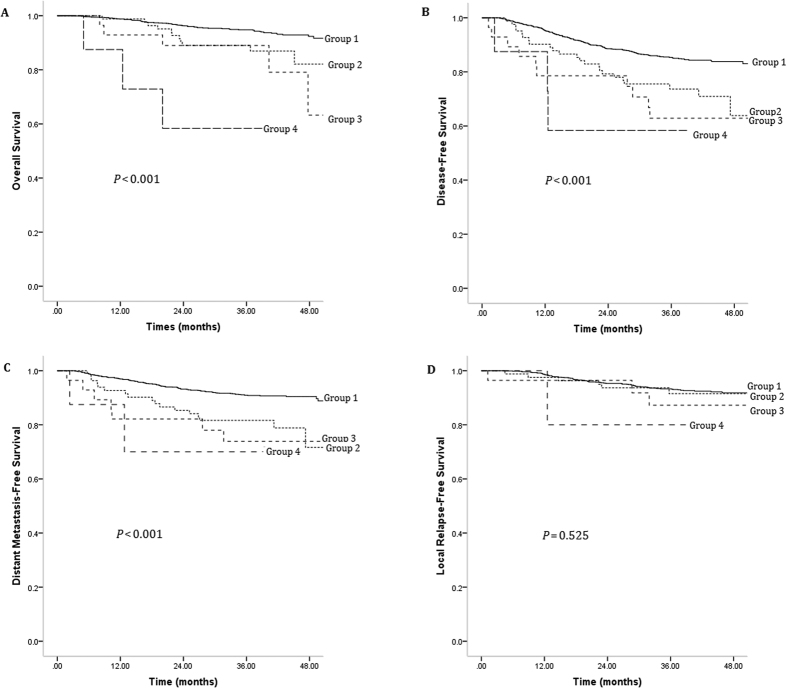
Kaplan–Meier survival curves showing overall survival rates (**A**), disease-free survival rates (**B**), distant metastasis–free survival rates (**C**) and local relapse–free survival rates (**D**) in nasopharyngeal carcinoma patients grouped according to pre- and post-treatment serum levels of lactate dehydrogenase (LDH). Group 1: normal pre- and post-treatment serum LDH levels, group 2: high pre-treatment and normal post-treatment plasma LDH levels, group 3: normal pre-treatment and high post-treatment plasma LDH levels, and group 4: high pre- and post-treatment plasma LDH level.

Patients were split into the following categories according to the baseline plasma LDH level and the change in LDH level after therapy (Table 2): group 1, normal pre- and post-treatment plasma LDH (1,309 patients); group 2, high pre-treatment and normal post-treatment plasma LDH (83 patients); group 3, normal pre-treatment and high post-treatment plasma LDH (28 patients); and group 4, high pre- and post-treatment plasma LDH (8 patients). The above patient groups were significantly correlated with the clinical stage (*P* = 0.025, Table 2). The 4-year OS rates in groups 1–4 were 92.4%, 82.1%, 63.3% and 58.3%, respectively (*P* < 0.001; Fig. 2A). Significant differences between the four subgroups were also seen in the case of the 4-year DFS (90.4%, 71.7%, 73.9% and 70.0%, respectively, *P* < 0.001; Fig. 2B) and 4-year DMFS (83.8%, 63.8%, 62.9% and 58.3%, respectively, *P* < 0.001; Fig. 2C). However, the 4-year LRFS did not differ between the four groups (91.8%, 91.5%, 87.2% and 80.0%, respectively, *P* = 0.525; Fig. 2D).

The paired-samples *t*-test revealed that the LDH level significantly declined after treatment (*P* < 0.001). The median and mean difference in the pre- and post-treatment LDH levels was 16 and 18.67 ± 1.60 IU/l. To evaluate the changes in LDH levels after treatment, we divided the patients into two groups according to the median difference: patients in whom the median decrease in LDH level after treatment was >16 IU/l and patients in whom this decrease was <16 IU/l or in whom there was an increase the LDH level after treatment. Patients in the former group had a 4-year OS of 92.8%, which was significantly better than survival rate in the latter group (88.9%, *P* = 0.007). Similarly, the DMFS rates in the two groups were significantly different (91.5% vs. 86.3% respectively, *P* = 0.005). The difference in DFS was only marginally significant (84.3% vs. 79.9%, respectively, *P* = 0.058), while no difference was detected in LRFS between the two groups (91.6% vs. 91.7%, *P* = 0.471).

### Multivariate analysis

Multivariate analysis was performed to adjust for various prognostic factors. The following parameters were included in a Cox proportional hazards model: age (<44 years vs. ≥44 years), sex, T classification, N classification, chemotherapy (present vs. absent), pre-treatment LDH levels (≤245 IU/l vs. >245 IU/l) and post-treatment LDH levels (≤245 IU/l vs. >245 IU/l). The N category, T category, post-treatment serum LDH levels and age were found to be independent prognostic factors for OS (*P* < 0.001, *P* < 0.001, *P* = 0.002 and *P* = 0.040 respectively). The multivariate analysis also revealed that the N category, T category and pre- and post-treatment serum LDH levels were significant predictors of DFS and DMFS. However, only the WHO histological type was an independent indicator of LRFS (Table 3).

## Discussion

Several prognostic variables, including LDH, have been identified to predict survival in NPC^8^,^13^. While the prognostic value of LDH levels at the time of diagnosis is well established, little is known about the value of post-treatment LDH levels as a marker of disease response. In the current study, we determined the pre- and post-treatment LDH levels in a cohort of NPC patients in order to determine the prognostic value of LDH as a dynamic parameter.

The results of our study showed that an elevated baseline LDH level was associated with distant failure and disease-specific survival. Many studies have shown that the LDH level at diagnosis is of prognostic significance in NPC, which was confirmed in the present study^4^,^7^,^8^,^14^. Liaw *et al*. retrospectively investigated 465 NPC patients and demonstrated that serum LDH levels were higher in patients who had distant metastasis or disease relapse than in patients who were in remission and in normal controls^4^. A phase III randomized study from Hong Kong compared the outcomes of concurrent chemoradiation versus radiation therapy alone in 348 NPC patients with advanced nodal disease, and found that serum LDH was a significant predictor of distant failure^15^. On the basis of these reports, we concluded that the baseline serum LDH level is a useful prognostic factor for NPC.

Although the prognostic significance of LDH in the case of OS, DFS and DMFS has been confirmed, the value of pretreatment LDH in predicting local relapse varies between studies^4^,^7^. In the current study, LDH level was not a significant factor for locoregional control on univariate and multivariate analyses. This result was consistent with that of our previous study, which included 465 NPC patients treated with IMRT^6^. However, Wan *et al*. have reported that among a cohort of NPC patients from a randomized controlled trial, pretreatment serum samples were a prognostic predictor of LRFS^7^. Moreover, in a prognostic scoring system for locoregional control proposed by Cheng *et al*., pretreatment LDH level was identified as an important parameter for LRFS prediction in NPC patients^9^. This difference among our study and the above studies may be attributable to differences in the treatments administered; Wan *et al*. and Cheng *et al*. used conventional two-dimensional radiotherapy, while our study involved IMRT. Compared with conventional radiotherapy, IMRT generates more conformal dose coverage in the target volume and therefore results in ideal local control^16^. In the current study, the 4-year LRFS was as high as 91.7%. Thus, it is possible that the improved local control rates obtained with IMRT eliminated the prognostic impact of LDH levels on LRFS.

The prognosis of a cancer depends on the biological aggressiveness of the tumor, the characteristics of the host and the therapeutic interventions. The prognostic factors of NPC have been an important research focus, and numerous investigations in this area have been published. Among the suggested disease-related prognosticators, the following have been proved to be significant prognostic factors in NPC: stage at diagnosis, plasma/serum Epstein–Barr virus DNA, serum LDH, tumor volume, cranial nerve palsy and the presence of tumor-associated genes in the peripheral blood cells^17^,^18^,^19^,^20^. However, the application of these pretreatment markers in clinical practice with consequent adjustments of the treatment regimen has never been shown to be feasible. Therefore, the search for new markers that can be used to predict and monitor early tumor-treatment responses will greatly impact patient management.

Our study suggests that post-treatment serum LDH levels were an independent prognosticator. Although it wasn’t the unique prognosticator, it indicated that besides the familiar risk factors such as TNM categories and age at diagnosis, the dynamic LDH levels during the treatment period should be considered, especially to define patients with a high risk of metastasis who warrant more aggressive systemic therapy to improve long-term outcomes when considering the addition of adjuvant or maintenance therapy at the end of radiotherapy. This is the first time that the prognostic value of post-treatment serum LDH levels has been explored in patients with newly diagnosed NPC treated with radical IMRT. Our conclusion was consistent with that of another study conducted in a cohort of patients with metastatic NPC. In 2012, Jin *et al*. reported that patients with elevated post-treatment LDH levels had worse survival than those with normal LDH levels. In their study, patients with normal pre- and post-treatment LDH values showed the highest response rate and the most favorable prognosis^21^. Notably, on the basis of our data, we recommend that LDH levels should be evaluated in NPC patients after the finish of radiotherapy. An increase in LDH at this time should lead to a thorough reevaluation of the disease status.

Serum LDH is potentially an ideal prognosticator among NPC patients treated with radical IMRT. During the search for prognostic indicators, a strong emphasis must be paid to ensure that the indicator is useful for both clinicians and researchers, while also being applicable in highly endemic regions all over the world. The convenience, repeatability and low cost of LDH assessments make this factor one of the most applicable in clinical practice. Furthermore, the prognostic indicator is important for not only predicting outcomes but also determining the proper management strategy for NPC patients. Since distant failure continues to be a critical point affecting patient prognosis, prognostic factors should focus on the risk of distant failure. Elevated pre- and/or post- treatment serum LDH levels predict an increased risk of both distant failure and suboptimal survival. More intensive systemic treatment strategies that can be used in this group of patients must be investigated.

This study identified that pre- and post-treatment LDH levels are negative prognostic indicators in NPC. High LDH is of great clinical significance in many other solid tumors as well as hematological malignancies^22^,^23^. However, the exact mechanism underlying this phenomenon is not understood. High serum LDH levels usually indicate the presence of a hypoxic environment associated with a large tumor burden^24^, and the oxygenation status of a tumor has been shown to be an important determinant of clinical outcomes among patients who receive radiotherapy and chemotherapy^25^. Alternatively, it was reported recently that increased LDH may reflect aberrant oncogene activity, since it is known that the myc and PI3K/Akt/mTOR pathways regulate cellular LDH expression levels^26^. The activation of these pathways was demonstrated to be an unfavorable prognostic factor in NPC^27^. Finally, LDH may be related to host factors, such as cellular turnover, cachexia and inflammation due to progressive tumor growth^22^.

To the best of our knowledge, this analysis is the first to demonstrate the prognostic role of dynamic serum LDH changes in patients with newly diagnosed, non-metastatic NPC treated with radical IMRT. Elevated pre- and post-treatment serum LDH levels were confirmed to be useful predictors of distant metastasis and poor overall survival. Thus, we strongly recommend that LDH determinations be carried out in NPC patients before and after radiotherapy, and that the test results be integrated into decisions regarding treatment strategy.

## Additional Information

**How to cite this article**: Zhou, G.-Q. *et al.* Prognostic implications of dynamic serum lactate dehydrogenase assessments in nasopharyngeal carcinoma patients treated with intensity-modulated radiotherapy. *Sci. Rep.* **6**, 22326; doi: 10.1038/srep22326 (2016).

## Figures and Tables

**Table 1:** Demographic and baseline characteristics of the study patients.

Characteristics	No. of patients (%) (*N* = 1428)	%
Age, years		
<44	729	51.0%
≥44	699	49.0%
Gender		
Male	1066	74.6%
Female	362	25.4%
Histological type		
WHO Type I	7	0.5%
WHO Type IIa/IIb	1421	99.5%
T category*		
T1	232	16.2%
T2	206	14.4%
T3	693	48.5%
T4	297	20.8%
N category*		
N0	219	15.3%
N1	852	59.7%
N2	221	15.5%
N3	136	9.6%
Clinical stage group*		
I	63	4.4%
II	277	19.4%
III	684	47.9%
IVA-B	404	28.3%
Chemotherapy		
CRT	150	10.5%
RT alone	1278	89.5%
Pre-treatment LDH		
≤245 IU/L	1339	93.8%
>245 IU/L	89	6.2%
Post-treatment LDH		
≤245 IU/L	1393	97.5%
>245 IU/L	35	2.5%

WHO, World Health Organization; CRT, chemoradiotherapy; RT, radiotherapy; LDH, lactate dehydrogenase; IU, international unit.

*According to the American Joint Committee on Cancer, 7th edition.

**Table 2:** Subgroups of patients according to the pre- and post-treatment serum LDH levels.

	Cases (n)	Pretreatment serum LDH (IU/L)			Post-treatment serum LDH (IU/L)			Clinical stage* (%)				*P*^*#*^
		Mean ± SD	Median	Range		Median	Range	I	II	III	IV	
Group 1	1309	168.6 ± 28.9	166.0	84.0–245.0	153.5 ± 29.1	151.0	16.0–244.0	62 (4.7%)	260 (19.9%)	634 (48.4%)	353 (27.0%)	0.025
Group 2	83	307.6 ± 87.7	274.0	246.0–753.0	176.9 ± 34.9	174.0	110.0–243.0	0 (0.0%)	11 (13.3%)	33 (39.8%)	39 (47.0%)	
Group 3	28	183.9 ± 26.8	191.5	140.0–241.0	285.4 ± 51.7	269.0	247.0–475.0	1 (3.0%)	5 (17.9%)	13 (46.4%)	9 (32.1%)	
Group 4	8	367.3 ± 134.5	313.0	251.0–565.0	508.7 ± 517.5	271.0	245.0–1756.0	0 (0%)	1 (12.5%)	4 (50%)	3 (37.5%)	

LDH, lactate dehydrogenase; IU, international unit.

*According to the American Joint Committee on Cancer, 7th edition.

^***#***^*P* values were calculated using the chi-square test.

**Table 3:** Multivariate analysis of prognostic factors for patients with nasopharyngeal carcinoma.

Endpoint	Variable	B	*P*-value	Exp(B)	95% CI for Exp(B)
Death	N category	0.526	0.000	1.692	1.454–1.969
	T category	0.509	0.000	1.663	1.295–2.137
	Post-treatment LDH	1.259	0.002	3.522	1.606–7.723
	Age	0.427	0.040	1.533	1.019–2.306
Disease progress	N category	0.339	0.000	1.404	1.260–1.563
	T category	0.215	0.004	1.240	1.073–1.432
	Post-treatment LDH	0.831	0.005	2.296	1.278–4.127
	Pre-treatment LDH	0.454	0.032	1.575	1.039–2.388
Distant failure^*^	N category	0.421	0.000	1.523	1.336–1.737
	T category	0.270	0.005	1.310	1.085–1.582
	Post-treatment LDH	0.979	0.005	2.662	1.347–5.264
	Pre-treatment LDH	0.571	0.023	1.770	1.082–2.894
Local failure	WHO histological type	−0.744	0.004	0.475	0.285–0.792

CI, confidence interval; LDH, lactate dehydrogenase; WHO, World Health Organization.

^*^According to the 7^th^ AJCC/UICC staging system.
